# Effects of Dairy Manure-Based Amendments and Soil Texture on Lettuce- and Radish-Associated Microbiota and Resistomes

**DOI:** 10.1128/mSphere.00239-19

**Published:** 2019-05-08

**Authors:** Giselle K. P. Guron, Gustavo Arango-Argoty, Liqing Zhang, Amy Pruden, Monica A. Ponder

**Affiliations:** a Via Department of Civil and Environmental Engineering, Virginia Tech, Blacksburg, Virginia, USA; b Department of Food Science and Technology, Virginia Tech, Blacksburg, Virginia, USA; c Department of Computer Science, Virginia Tech, Blacksburg, Virginia, USA; University of Wisconsin—Madison

**Keywords:** agriculture, antibiotic resistance, compost, manure, resistome, soil, vegetable microbiome

## Abstract

Dairy cattle are routinely treated with antibiotics, and the resulting manure or composted manure is commonly used as a soil amendment for crop production, raising questions regarding the potential for antibiotic resistance to propagate from “farm to fork.” The objective of this study was to compare the microbiota and “resistomes” (i.e., carriage of antibiotic resistance genes [ARGs]) associated with lettuce leaf and radish taproot surfaces grown in different soils amended with dairy manure, compost, or chemical fertilizer only (control). Manure was collected from antibiotic-free dairy cattle (DC) or antibiotic-treated dairy cattle (DA), with a portion composted for parallel comparison. Amendments were applied to loamy sand or silty clay loam, and lettuce and radishes were cultivated to maturity in a greenhouse. Metagenomes were profiled via shotgun Illumina sequencing. Radishes carried a distinct ARG composition compared to that of lettuce, with greater relative abundance of total ARGs. Taxonomic species richness was also greater for radishes by 1.5-fold. The resistomes of lettuce grown with DC compost were distinct from those grown with DA compost, DC manure, or fertilizer only. Further, compost applied to loamy sand resulted in twofold-greater relative abundance of total ARGs on lettuce than when applied to silty clay loam. The resistomes of radishes grown with biological amendments were distinct from the corresponding fertilizer controls, but effects of composting or antibiotic use were not measureable. Cultivation in loamy sand resulted in higher species richness for both lettuce and radishes than when grown in silty clay loam by 2.2-fold and 1.2-fold, respectively, when amended with compost.

**IMPORTANCE** A controlled, integrated, and replicated greenhouse study, along with comprehensive metagenomic analysis, revealed that multiple preharvest factors, including antibiotic use during manure collection, composting, biological soil amendment, and soil type, influence vegetable-borne resistomes. Here, radishes, a root vegetable, carried a greater load of ARGs and species richness than lettuce, a leafy vegetable. However, the lettuce resistome was more noticeably influenced by upstream antibiotic use and composting. Network analysis indicated that cooccurring ARGs and mobile genetic elements were almost exclusively associated with conditions receiving raw manure amendments, suggesting that composting could alleviate the mobility of manure-derived resistance traits. Effects of preharvest factors on associated microbiota and resistomes of vegetables eaten raw are worthy of further examination in terms of potential influence on human microbiomes and spread of antibiotic resistance. This research takes a step toward identifying on-farm management practices that can help mitigate the spread of agricultural sources of antibiotic resistance.

## Abstract (precis)

A controlled, integrated, and replicated greenhouse study, along with comprehensive metagenomic analysis, revealed that multiple preharvest factors, including antibiotic use during manure collection, composting, biological soil amendment, and soil type, influence vegetable-borne resistomes. Here, radishes, a root vegetable, carried a greater load of ARGs and species richness than lettuce, a leafy vegetable. However, the lettuce resistome was more noticeably influenced by upstream antibiotic use and composting. Network analysis indicated that cooccurring ARGs and mobile genetic elements were almost exclusively associated with conditions receiving raw manure amendments, suggesting that composting could alleviate the mobility of manure-derived resistance traits. Effects of preharvest factors on associated microbiota and resistomes of vegetables eaten raw are worthy of further examination in terms of potential influence on human microbiomes and spread of antibiotic resistance. This research takes a step toward identifying on-farm management practices that can help mitigate the spread of agricultural sources of antibiotic resistance.

## INTRODUCTION

Worldwide administration of antimicrobial agents to livestock is projected to rise from more than 60,000 tons in 2010 to more than 105,000 tons by 2030 (1). It is well established that antibiotic administration can increase levels of antibiotic-resistant bacteria (ARB) or antibiotic resistance genes (ARGs) in manure (2, 3). Residual antibiotics and metabolites are also excreted in urine and feces (4, 5), which can result in proliferation of ARB and ARGs in soil after application (6–8). Soil is a known source of microbiota colonizing plants, such as tomatoes and grapevines (9, 10), and the soil and soil amendments have been observed to share ARGs, such as *TEM* and *aadA*, in common with cultivated *Brassica* plants (11). Thus, both soil and associated amendments can seed and colonize not only root vegetables, where there is direct contact, but also portions of the crop that are physically disparate from the soil, presumably through suspension and transfer of soil material or motility of microorganisms colonizing roots.

Transfer of ARB from environmental sources to the surfaces of fruits and vegetables, especially those eaten raw, could act as a bridge between environmental ARB/ARGs and those acquired by human pathogens or harbored in the gut microbiota. Tetracycline-resistant Escherichia coli isolated from retail salad greens also carried plasmids bearing ARGs encoding resistance to beta-lactams, tetracyclines, and quinolones (12). Gram-negative ARB carrying integron-associated ARGs, such as *mcr-1* and *TEM-1*, have been identified on ready-to-eat produce in Portugal (13). Thus, there is a need to understand how various preharvest practices, such as application of manure-based amendments derived from cattle receiving antibiotics, will influence the variety of ARB and ARGs occurring on surfaces of fresh fruits and vegetables. Rather than focus on single strains or pathogens, a broad understanding of factors that mediate the potential for ARB and ARGs to evolve and spread through each stage of vegetable production is called for (14). A holistic “resistome” (15) framework in which the types and abundances of ARGs and gene transfer elements are comprehensively profiled via shotgun metagenomic sequencing holds promise for achieving this purpose.

The main objective of this study was to profile and compare the metagenomes, including microbiota and resistomes, of greenhouse-grown lettuce leaves and radish taproots cultivated with different soil types (loamy sand [LS] and silty clay loam [SCL]) amended with manure from dairy cattle collected with typical administration of antibiotics (pirlimycin and cephapirin) (DA) and without typical administration of antibiotics (DC) or corresponding composts, compared to chemical-fertilizer-only controls. The hypothesis was that antibiotic use in livestock generating manure would alter the resistome of vegetables grown with corresponding amendments but that composting and soil type would mediate the effects. ARGs were identified through comparison to the Comprehensive ARG Database (CARD) (16), using commonly applied cutoff criteria, and also to ARG-miner (17), a recently introduced web-based tool that combines multiple publicly available databases (including CARD) along with a deep learning algorithm (18) for the purpose of avoiding false-negative results and maximizing identification of putative, novel ARGs. Genetic markers of horizontal gene transfer colocalized with ARGs were identified through assembly and network analysis to gain insight into potential mechanisms of ARG proliferation. The greenhouse experimental design enabled a highly integrative study; simultaneously examining the effects of antibiotic use in livestock, manure treatment, biological soil amendment, soil texture, and vegetable type. This provided an ideal means to compare the relative contributions of these factors toward shaping vegetable microbiomes and informing optimal management practices for reducing the potential for farm-to-fork transmission of antibiotic resistance.

## RESULTS

### Identification of ARGs using CARD and ARG-miner.

A total of 871,651,806 DNA reads were generated across 50 samples, with an average of 17,433,036 reads per sample (see Table S1 in the supplemental material). 16S rRNA gene sequences obtained from the lettuce and radish shotgun libraries were found to be 50.8 to 94.5% and 0.0 to 5.2% derived from chloroplasts, respectively. Libraries were rarefied to 12,700,000 reads, with chloroplast 16S rRNA gene sequences removed to facilitate statistical comparison. Given that metagenomic sequencing provides a relative comparison, all annotated ARGs were normalized to bacterial 16S rRNA genes as an indicator of the proportion of the bacterial community carrying ARGs and potential selection pressure at play (19). There were no remarkable differences between pooled and individually sequenced samples (see Text S1 in the supplemental material), and subsequent analyses considered the pooled samples as individual replicates.

10.1128/mSphere.00239-19.1 TEXT S1 Supplemental results and discussion. Download Text S1, DOCX file, 0.01 MB. Copyright © 2019 Guron et al. 2019 Guron et al. This content is distributed under the terms of the Creative Commons Attribution 4.0 International license.

10.1128/mSphere.00239-19.3 TABLE S1 Samples sequenced for this study. Lettuce and radishes were grown in soils amended with chemical fertilizer only or biological amendments derived from antibiotic-treated cattle (DA) or antibiotic-free cattle (DC). The high-quality reads generated, number of reads that aligned to specified databases, and the assembly quality are also summarized. Number of reads identified from CARD v1.2.1 are prior to removing “housekeeping” genes from the data set (Table S8). Download Table S1, TXT file, 0.01 MB. Copyright © 2019 Guron et al. 2019 Guron et al. This content is distributed under the terms of the Creative Commons Attribution 4.0 International license.

**FIG 1:**
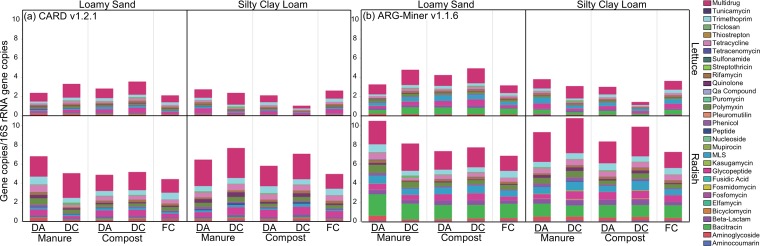
Mean relative abundance of total DNA sequence reads annotated as ARGs. Thirty-one different antibiotic resistance classes were represented, normalized to the number of 16S rRNA gene copies (excluding sequences from chloroplasts) of each treatment type for lettuce leaves or radish taproots as aligned to CARD v1.2.1 (E value < 1e−10, 80% identity, ≥25 amino acids) (a) or ARG-miner v1.1.6 (b). Each sample was rarefied to 12,700,000 reads. Vegetables were grown in loamy sand (LS) with dairy antibiotic (DA) or dairy control (DC) manure or compost (*n* = 2 for each amendment type in LS), in silty clay loam (SCL) with DA or DC manure or compost (*n* = 3 for each amendment type in SCL), in chemical-fertilizer-only control LS (*n* = 2), or in chemical-fertilizer-only control SCL (*n* = 3). Housekeeping genes present in CARD were excluded from this analysis (see Table S8 in the supplemental material).

The consistencies in trends using the cutoff criteria/CARD versus ARG-miner pipelines for characterizing the effect of amendment type for each soil texture on vegetable-borne ARGs were notable (Fig. 1). An average of 1.4- and 1.5-fold-greater ARGs were detected on lettuce and radishes by ARG-miner, respectively, compared to when CARD was used. Across all samples, 60,224 lettuce-associated reads and 194,787 radish-associated reads aligned to 614 and 682 ARGs from CARD, respectively, conferring resistance to 23 classes of antibiotics (Fig. 1a; see also Table S2). Likewise, 79,920 lettuce-associated reads and 282,336 radish-associated reads aligned to 3,249 and 3,826 genes, respectively, from the ARG-miner pipeline, among 26 classes of antibiotics (Fig. 1b; see also Table S3). There were 406 and 450 ARGs from lettuce and radishes, respectively, detected by both pipelines, with the four most common classes being multidrug, macrolide-lincosamide-streptogramin (MLS), beta-lactam, and aminoglycoside classes. For lettuce, 47.7% of reads aligning to CARD and 40.8% of reads aligning to ARG-miner corresponded to multidrug ARGs. For radishes, 52.7% of reads aligning to CARD and 40.9% of reads aligning to ARG-miner corresponded to multidrug ARGs. Additional comparisons of the CARD and ARG-miner annotations are provided in the supplemental material (Text S1). Subsequent results focus on CARD annotations.

10.1128/mSphere.00239-19.4 TABLE S2 MetaStorm output of ARGs from CARD. Relative abundance of ARGs annotated using CARD v1.2.1 normalized to 16S rRNA gene copies after exclusion of housekeeping genes and rarefaction to 12,700,000 reads. Zero values indicate genes below detection. Download Table S2, TXT file, 0.3 MB. Copyright © 2019 Guron et al. 2019 Guron et al. This content is distributed under the terms of the Creative Commons Attribution 4.0 International license.

10.1128/mSphere.00239-19.5 TABLE S3 MetaStorm output of ARGs from ARG-Miner. Relative abundance of ARGs annotated using ARG-miner normalized to 16S rRNA gene copies after exclusion of housekeeping genes and rarefaction to 12,700,000 reads. Zero values indicate genes below detection. Download Table S3, TXT file, 0.9 MB. Copyright © 2019 Guron et al. 2019 Guron et al. This content is distributed under the terms of the Creative Commons Attribution 4.0 International license.

**FIG 2:**
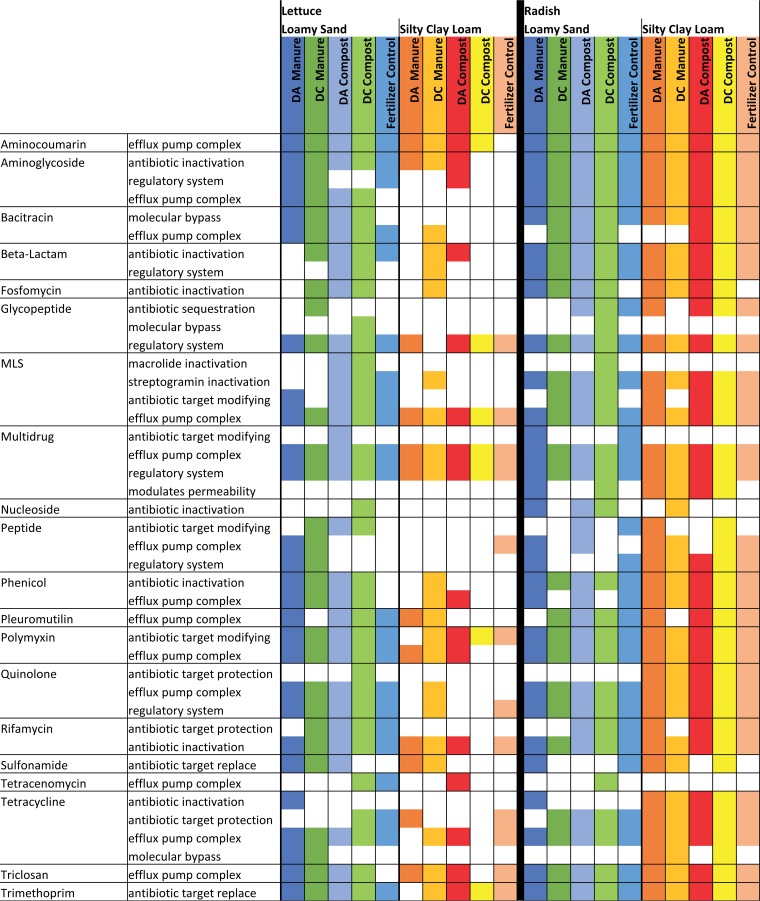
Detection (colored) or nondetection (white) of ARGs corresponding to specific ARG mechanisms. Mechanisms were determined using assembled sequences aligned to CARD v1.2.1 (E value < 1e−10, 60% identity, ≥25 amino acids). Both vegetables were grown in two soil textures with biological amendments from antibiotic-treated cattle (DA) or antibiotic-free cattle (DC). Vegetables were grown in loamy sand (LS) with dairy antibiotic (DA) or dairy control (DC) manure or compost (*n* = 2 for each amendment type in LS), in silty clay loam (SCL) with DA or DC manure or compost (*n* = 3 for each amendment type in SCL), in chemical-fertilizer-only control LS (*n* = 2), or in chemical-fertilizer-only control SCL (*n* = 3). Note that data are presented as presence/absence because although assembled data have higher resolution, they lose quantitative information.

After assembling the reads to obtain higher functional resolution, a total of 42 specific resistance mechanisms subcategorized across 20 antibiotic classes were identified through comparison to CARD (Fig. 2). The number of antibiotic resistance mechanisms detected was dependent on vegetable type when analyzed across all SCL conditions (*P* < 0.040), but not when analyzed across all LS conditions. Additionally, the number of antibiotic resistance mechanisms detected on lettuce was dependent on the amendment type when grown in SCL (*P* < 0.009), but not when lettuce was grown in LS or for radishes grown in either soil texture. Antibiotic resistance mechanisms on lettuce were also dependent on soil texture when analyzed across DA manure and both composts (*P* < 0.045), but there were no soil effects across any amendment types for radishes.

### Comparison of lettuce and radish resistomes.

To determine how direct contact with soil may influence the corresponding resistome of the edible vegetable portions, lettuce was selected as a leafy vegetable, while radish was selected as a root vegetable. Notably, lettuce versus radish ARG profiles were markedly distinct (global *R* = 0.760) when comparing at the class level (*P* < 0.001). Radishes carried an average load of 2.4-fold-greater total ARGs than lettuce (*P* < 0.001), which largely reflected more than threefold differences in beta-lactam, fosfomycin, phenicol, polymyxin, and quinolone classes (*P* < 0.001). In terms of diversity of ARGs encountered, radishes carried 1.4-fold-greater richness than lettuce (*P* < 0.001) (Table S4). Lettuce carried 2.9-fold-greater sulfonamide ARGs than radishes (*P* < 0.001). Differences were less apparent at the class level with 23 ARG classes detected for each based on comparison to CARD.

10.1128/mSphere.00239-19.6 TABLE S4 Alpha diversity of ARGs and OTUs. ARGs were detected using CARD v1.2.1 (E value < 1e−10, 80% identity, ≥25 amino acids), and taxa were identified using MetaPhlAn2 after rarefaction to 12,700,000 reads. Richness indicates the number of ARGs detected, H_max_ = natural log of richness, Shannon (H’) = −∑[(n1/N)ln(n1/N)], Evenness = H’/H_max_. Download Table S4, DOCX file, 0.03 MB. Copyright © 2019 Guron et al. 2019 Guron et al. This content is distributed under the terms of the Creative Commons Attribution 4.0 International license.

### Effects of biological amendments and soil textures on lettuce resistomes.

**FIG 3:**
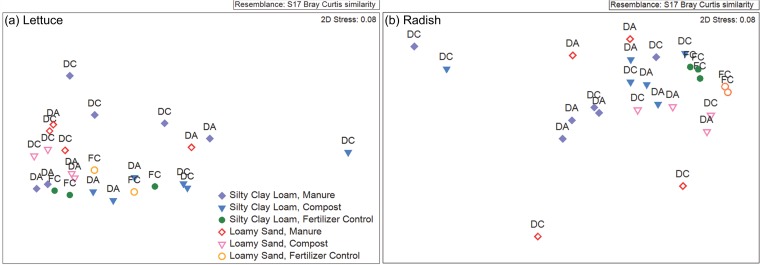
Nonmetric multidimensional scaling plot based on vegetable ARGs. Lettuce leaves (a) and radish taproots (b) were grown in each soil texture mixed with manure, compost, or fertilizer control based on Bray-Curtis similarities calculated from ARG relative abundances. Each sample was rarefied to 12,700,000 reads, and chloroplast 16S rRNA gene sequences were excluded. Biological amendments were generated from antibiotic-treated cattle (DA) or antibiotic-free cattle (DC). Genes included in the analysis for this study and the housekeeping genes present in CARD that were excluded are summarized in Table S8.

For lettuce, both collecting manure during antibiotic administration and composting prior to application to the soil resulted in slightly different profiles of ARG classes (global *R* = 0.298, *P* < 0.013) (Fig. 3a). Specifically, the ARGs carried by lettuce grown in soils with DC compost were distinct in composition relative to those of lettuce grown in soils with DA compost (*R* = 0.629, *P* < 0.033), DC manure (*R* = 0.543, *P* < 0.033), or chemical fertilizer only (*R* = 0.629, *P* < 0.033). Lettuce ARG profiles were also distinct when grown in DA compost- versus DC manure-amended soil (*R* = 0.657, *P* < 0.033). Soil texture was also associated with some distinction in the lettuce ARG profiles (*R* = 0.350, *P* < 0.044).

There was no statistically significant difference in the total relative abundances of ARGs carried by lettuce among the soil or amendment conditions tested. However, there were significant differences when comparing relative abundances of specific antibiotic resistance classes. Specifically, differences were noted for lettuce grown in SCL when the DA/DC manure conditions were combined to represent a “manure” condition and compared to the combined DA/DC “compost” conditions: aminoglycoside (*P* < 0.007) and triclosan ARGs (*P* < 0.013) were greater by 2.3-fold and 11.1-fold, respectively, indicating an overall effect of composting for this soil type. No differences in individual ARG class abundances were noted in the LS soil condition.

Soil texture had an overarching and significant effect on the profiles of ARGs carried by lettuce (*R* = 0.350, *P* < 0.044) (Fig. 2a). The combined effects of compost and soil texture were particularly notable, e.g., total ARGs trended lowest among all conditions, even fertilizer control, when DC compost was added to SCL, but not to LS (twofold difference; *P* < 0.010) (Fig. 1a). Further, lettuce grown in compost-amended LS carried 2.2-fold-greater diversity (richness) in ARGs than the corresponding SCL condition (*P* < 0.010). Specific ARG classes that were greater in the compost-amended LS condition were aminoglycoside (2.4-fold), bacitracin (5.1-fold), glycopeptide (1.7-fold), MLS (2.5-fold), multidrug (2.3-fold), pleuromutilin (2.6-fold), quinolone (2.9-fold), rifamycin (1.7-fold), and triclosan (11.5-fold) (*P* < 0.010). Soil textures for manured treatments affected only bacitracin ARGs on lettuce, which were 2.5-fold greater in LS than SCL (*P* < 0.038). Overall, the results suggest some potential benefits of composting for reducing total ARGs and certain specific classes of ARGs carried on lettuce, but only for certain soil textures.

### Effects of biological amendments and soil textures on radish resistomes.

Unlike the lettuce leaf surfaces, radish ARG profiles from DA or DC compost-amended soils were not distinguishable (*R* = 0.171, *P* > 0.05), but addition of biological amendments (global *R* = 0.617, *P* < 0.001) and the soil texture (global *R* = 0.472, *P* < 0.001) did have a significant effect (Fig. 3b). In particular, the resistomes of radishes grown in fertilizer-only control soils were markedly distinct from radishes grown with DC manure (*R* = 0.457, *P* < 0.033), DA manure (*R* = 1.000, *P* < 0.033), DC compost (*R* = 0.600, *P* < 0.033), and DA compost (*R* = 0.971, *P* < 0.033). Additionally, DA compost was associated with a shift in the radish resistome compared to DA manure (*R* = 1.000, *P* < 0.033).

In terms of relative abundance of total ARGs on radish surfaces, there was no statistically significant effect of composting relative to raw manure (Fig. 1a). Still, the relative abundance of total ARGs trended highest for radishes grown in DA manure-amended LS relative to the other LS amendments, by more than 1.3-fold. The LS condition, where this trend was most noticeable, was limited to two replicates and would require greater statistical power to confirm. Individual ARG classes found to be significantly more abundant on radishes grown in compost-amended LS included pleuromutilin (1.8-fold, *P* < 0.010), sulfonamide (3.0-fold, *P* < 0.024), and tetracenomycin (2.0-fold, *P* < 0.038). Conversely, radishes grown in compost amended to SCL carried more ARGs belonging to aminocoumarin (1.9-fold, *P* < 0.038), quinolone (2.0-fold, *P* < 0.010), and trimethoprim (1.3-fold, *P* < 0.010) classes than when amended to LS. The effect of soil texture when manure was applied was apparent only for peptide ARGs, where they were 3.1-fold greater for SCL than LS (*P* < 0.019).

### Taxonomic profiling of lettuce and radish surficial microbiota.

Consistent with observations with respect to the resistome comparisons, the β-diversity of the microbial communities were highly distinct when comparing lettuce and radish (*R* = 0.948, *P* < 0.001) (see Fig. S1 in the supplemental material). Richness and Shannon index was also greater for radishes by 1.5-fold and 1.1-fold, respectively (*P* < 0.025) (Table S4). In terms of soil type, cultivation in LS resulted in higher species richness for both lettuce and radishes than when grown in SCL by 2.2-fold and 1.2-fold, respectively, when amended with compost (*P* < 0.019). While there was no significant effect of soil texture on the β-diversity of lettuce microbial communities (*R* = 0.287, *P* < 0.065), radish β-diversity was highly affected by soil texture (*R* = 0.982, *P* < 0.001).

10.1128/mSphere.00239-19.2 FIG S1 Nonmetric multidimensional scaling plot of vegetable-associated microbiota. Lettuce and radishes were grown in each soil mixed with manure, compost, or fertilizer-only control based on Bray-Curtis similarities of species profile identified by MetaPhlAn v2 pipeline. Biological amendments were generated from antibiotic-treated cattle (DA) or antibiotic-free cattle (DC). Download FIG S1, PDF file, 0.4 MB. Copyright © 2019 Guron et al. 2019 Guron et al. This content is distributed under the terms of the Creative Commons Attribution 4.0 International license.

Compared to the fertilizer control, lettuce microbiota were affected by DA manure (*R* = 0.368, *P* < 0.038), DC manure (*R* = 1, *P* < 0.033), and DC compost (*R* = 0.971, *P* < 0.033). Compared to DC compost, lettuce communities were also affected by DA manure (*R* = 0.532, *P* < 0.029), DC manure (*R* = 1, *P* < 0.033), and DA compost (*R* = 0.885, *P* < 0.033). For radishes, all amendments significantly affected the taxonomic composition relative to the fertilizer control (*R* > 0.857, *P* < 0.033). Individually, each of the biological amendments also resulted in distinct radish microbiota compared to each other (*R* > 0.520, *P* < 0.033), except DC versus DA manure amendment.

**FIG 4:**
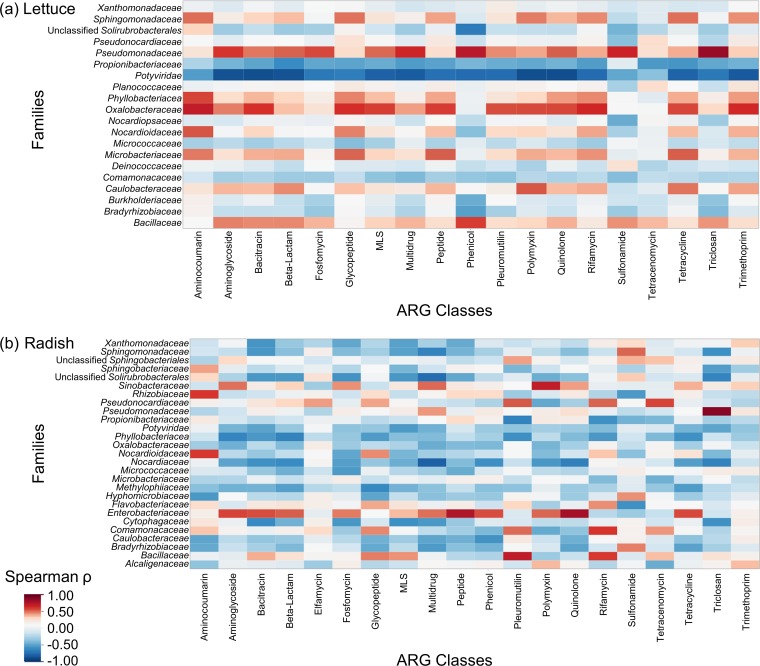
Spearman (ρ) correlations between taxonomic families and ARGs. Taxa were determined by MetaPhlAn2, and relative abundances of ARGs, as aligned to CARD v1.2.1, from lettuce leaves (a) or radish taproots (b). Each sample was rarefied to 12,700,000 reads, and chloroplast 16S rRNA gene sequences were excluded. Families and ARG classes that were detected in more than 75% of lettuce or radish samples were included. Red cells indicate positive correlations, while blue cells indicate negative correlations.

Both lettuce and radish were dominated by *Proteobacteria*, *Firmicutes*, and *Actinobacteria* (Table S5). Radishes were also dominated by *Bacteroides*, but this class was detected on only 52% of all lettuce. For both vegetables, *Pseudomonadaceae* were highly correlated with triclosan ARGs (ρ > 0.91) (Fig. 4). On radishes, Enterobacteriaceae were also highly correlated with quinolone ARGs (ρ = 0.82) and *Nocardiaceae* were negatively correlated with multidrug ARGs (ρ = −0.80).

10.1128/mSphere.00239-19.7 TABLE S5 Lettuce and radish families. The 20 most abundant families associated with lettuce and radish surfaces (reads per kilobase per million mapped reads). Vegetables were grown in loamy sand (LS) or silty clay loam with dairy antibiotic (DA) or dairy control (DC) manure, compost, or chemical fertilizer-only control. nd, values not detected. Download Table S5, DOCX file, 0.02 MB. Copyright © 2019 Guron et al. 2019 Guron et al. This content is distributed under the terms of the Creative Commons Attribution 4.0 International license.

### Occurrence patterns for ARGs of potential “clinical concern.”

Since DA cattle were treated with cephapirin and pirlimycin, a first-generation cephalosporin and lincosamide, respectively, beta-lactam and MLS ARGs were analyzed separately (Table S6). Unassembled rarefied reads were screened for occurrence of families of beta-lactamase genes from functional groups 1 and 2 that hydrolyze cephalosporins (20). *OXA*, *CTX-M*, *SHV*, *TEM, CMY, KPC, VEB, PER, GES,* and *SME* variants of *bla* ARG types were detected on lettuce and radishes (ranked high to low abundance across data sets). *OXA* was prevalent on surfaces of both vegetable types and across all conditions, but *OXA* was 1.5-fold greater on radishes than on lettuce (*P* < 0.032). Conversely, *TEM* was 2.7-fold greater on lettuce than on radishes (*P* < 0.001).

10.1128/mSphere.00239-19.8 TABLE S6 ARGs of critical concern. Average relative abundance from each vegetable treatment of certain gene families of antibiotics of critical concern after rarefaction to 12,700,000 reads and removal of chloroplast 16S rRNA gene sequences. nd, values not detected. Download Table S6, DOCX file, 0.02 MB. Copyright © 2019 Guron et al. 2019 Guron et al. This content is distributed under the terms of the Creative Commons Attribution 4.0 International license.

Genes for erythromycin ribosome methylation (*erm*) were also analyzed because modification of the 50S rRNA subunit causes cross-resistance to lincosamides (21, 22). *erm* genes were detected in all treatments for both vegetable types, with relative abundances ranging from 0.006 to 0.024 gene copies/16S rRNA for radishes grown in DA manure-amended LS versus lettuce grown in DC compost-amended LS, respectively. For compost-amended lettuce, *erm* genes were 2.1-fold greater when grown in LS than SCL (*P* < 0.010). Lincosamide inactivation genes, *lnu* (22), were also identified, but are not known to confer resistance more broadly to macrolides. Overall, *lnu* was 5.5-fold greater on lettuce than on radishes (*P* < 0.001).

Reads were also screened for ARGs corresponding to resistance to other “highest priority” antibiotics, as classified by the World Health Organization (23) (Table S6). Gene variants detected on both vegetables include mobilized colistin resistance (*mcr*) (24), quinolone resistance (*qnrB*) (25), and glycopeptide resistance gene clusters *vanHAX* (genes for dehydrogenase, D-Ala-D-Lac ligase, and D,D-dipeptidase, respectively) (26). *vanA* and *vanX* variants were 1.3-fold greater on radishes than on lettuce (*P* < 0.039). *vanA* and *vanX* variants were also 3.5-fold and 5.8-fold greater on compost-amended lettuce from LS than from SCL (*P* < 0.016). Only *vanA* variants were greater on manure-amended radishes by 3.3-fold when grown in SCL than in LS (*P* < 0.038).

### Assembly and network analysis confirm known gene arrangements and identify potentially mobile ARGs.

**FIG 5:**
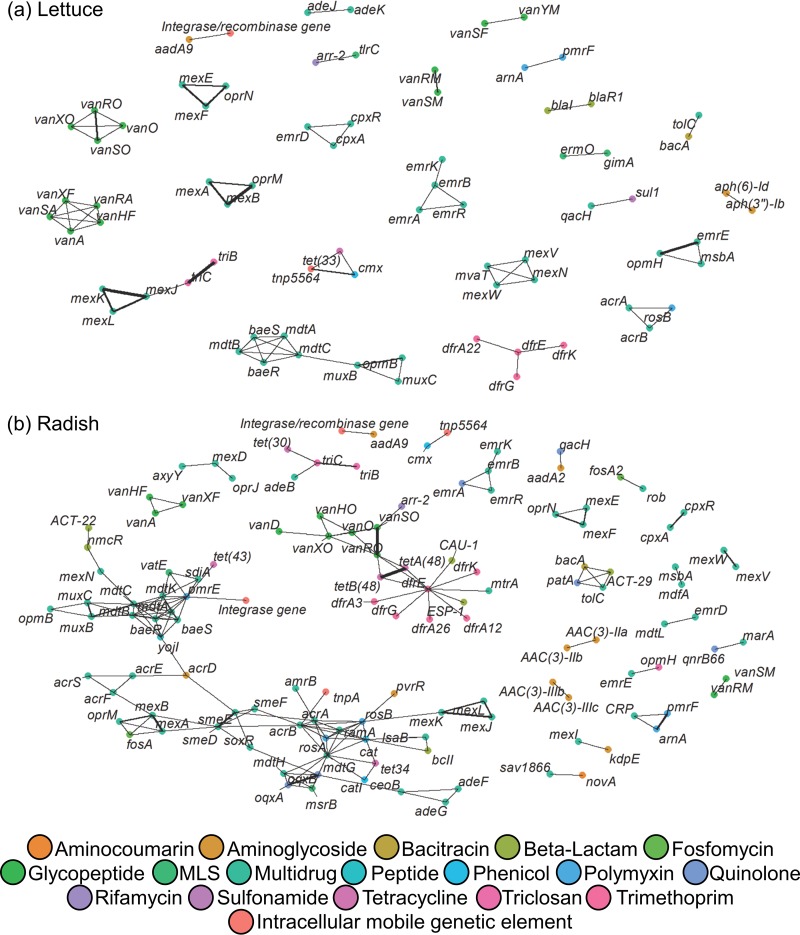
Cooccurrence of ARGs and putative intracellular mobile genetic elements. This network analysis maps out cooccurrences of ARGs with each other, as an indicator of potential for coselection, and with plasmid-associated genes, as an indicator of mobility. Network analysis highlights ARGs detected using CARD v1.2.1 (E value < 1e−10, 60% identity, ≥25 amino acids) that cooccur on the same scaffolds among all the lettuce leaf (a) or radish taproot (b) surfaces (*n* = 25). Each node represents a gene, and each edge connects the cooccurring nodes. Edge thickness indicates how often the cooccurring genes assembled among all metagenome sequences.

ARGs predicted to cooccur with each other and with mobile genetic elements (Fig. 5) were of particular interest as indicators of coresistance and mobility. Seventy-four ARGs representing 13 classes were identified as cooccurring on the same scaffolds generated from lettuce leaf surfaces, with most corresponding to multidrug resistance (Fig. 5a). Among radishes, more scaffolds and greater n50 lengths were successfully generated, by 1.2-fold and 1.6-fold, respectively (*P* < 0.035) (Table S1), resulting in identification of 120 ARGs across 16 ARG classes occurring on the same scaffolds (Fig. 5b). These mostly corresponded to multidrug and related regulatory genes for both lettuce (37/74 ARGs) and radishes (60/120 ARGs).

Genetic elements that mapped together with ARGs included the following: *cmx* (chloramphenicol) downstream of transposase *tnp5564* (GenBank accession no. AY266269.1; 99% identity); partial *aadA9* (aminoglycoside) downstream of an unspecified integrase gene (CP018135.1; >99% identity); *acrB* (multidrug efflux) (79% identity) downstream of transposase *tnpA* (96% identity) (CP022135.1). Remarkably, manure amendment was a commonality of all of these scaffolds containing ARGs and genetic elements associated with gene mobility. The only scaffold containing an ARG and mobile genetic element mapped from compost amendment was a *pmrE* upstream of an unspecified integrase gene (HG938354.1; 89% identity).

## DISCUSSION

Multiple databases and algorithms have been applied for identifying ARGs, including CARD Resistance Gene Identifier (16), ResFinder (27), MEGARes (28), and ARGs-OAP (29), each carrying their own assumptions and limitations. Here we compared alignment with the CARD database using defined cutoff criteria (16) and deep-learning-enabled ARG-miner (17). We highlight striking similarity in the overall trends and conclusions. This is consistent with findings where databases of different ARGs still yield similar results in terms of total ARG abundance (30). The higher ARG abundances estimated by ARG-miner were as expected, with the greater sensitivity of the deep-learning algorithm and ability to detect ARGs not yet reported in databases (17).

Vegetable type resulted in the strongest effect on the vegetable resistomes and the diversity of host-associated microbiota. Here, radishes carried a greater load and larger diversity of ARGs than lettuce, which may relate to their degree of contact with the soil, but plant species itself also shapes the overall rhizosphere and phyllosphere microbiota (31–33). Prior studies have examined ARGs on individual vegetables in terms of PCR-based methods, where different gene target profiles were detected among lettuce, radishes, and carrots (34–36). Metagenomics has been applied only to a limited degree to characterize field-grown carrot peel resistomes (34, 37). Studies examining the roles of plant species are of future interest, including distinctions between root versus leaf surfaces for the same plant type (9, 10). Considering degree of contact with soil could be a key factor in formulating future guidance toward mitigating potential for antibiotic resistance to spread from “farm to fork.”

The fact that soil was the next strongest factor shaping the resistome was surprising. To our knowledge, effects of soil texture on vegetable resistomes have not previously been reported. In this study, soil texture had a significant overarching effect on the resistome composition for both vegetables and further affected radish taxonomic β-diversity. Further study is needed to identify mechanistic aspects of how various soils interact with biological amendments to amplify or attenuate carriage of resistance elements. For example, addition of compost to LS actually resulted in higher carriage of ARGs by lettuce than when amended to SCL, which trended even lower than the fertilizer control. The combination of various abiotic factors, such as pH or charge, has been observed to affect soil microbial communities (31, 38). Differences in the beta-diversity of bacterial communities in tomato rhizosphere soil due to soil texture gradients have also been observed (39). On grapevines, the root zone bacterial community beta diversity is affected by soil pH and C/N ratio, while the beta-diversity of the bacterial communities of above-ground plant organs, such as the leaves of grapevines, are altered by soil carbon and moisture (10).

To our knowledge, no prior study has tracked the effects of antibiotic administration toward identifying corresponding ARG carriage of vegetables cultivated with corresponding manure-based amendments. The addition of biological amendments to the soil had a marked effect on ARG profiles of both vegetables relative to chemical fertilizer. Interestingly, though radishes had more direct contact with the soil and amendments, the antibiotic administration history had a more measureable effect on lettuce. This was observed both in terms of the ARG β-diversity and specifically when comparing the DA versus DC compost ARG profiles. Notably, composting had an effect on the resistomes of both vegetable types, e.g., resistomes of radishes amended with raw versus composted DA manure. Overall, the results suggest that composting alters resistomes, meriting additional investigation to determine whether this translates into mitigation of the risk of spreading antibiotic resistance.

Taxonomic analysis indicated that nearly all the different amendments applied here, including conditions with/without prior antibiotic use history, had a significant effect on the vegetable microbial community composition. This is a strong indicator that the experimental conditions were imposed on the vegetables as intended. The fact that effects on resistomes were observed to a lesser degree may reflect mobility of ARGs among the microbial communities. The positive correlations identified between taxonomic groups and ARGs were consistent with known ARG host ranges. Pseudomonas aeruginosa genome assemblies carry *triABC-opmH* (40), which confers resistance to triclosan (41). *qnr* determinants, encoding quinolone resistance, have been identified from Enterobacteriaceae (21). Notably, radishes carried both higher ARG and taxonomic β-diversity than lettuce, suggesting a potential relationship between microbial community and resistome diversity.

Digging deeper into the metagenomic data revealed notable differences in terms of profiles of ARGs (i.e., “resistomes”), ARG diversity, and responses of specific ARG classes and types. This highlights the potential to mine metagenomic data as a monitoring and assessment tool. One approach may be to search for specific ARG classes corresponding to critically important antibiotics (14). In the present study, several cephalosporin, MLS, colistin, quinolone, and glycopeptide ARGs were found, with some detectable in all fertilizer controls (e.g., *OXA*, *SHV*, *erm*, and *vanHAX* variants), whereas others (*KPC*, *qnrB*, and *qnrD*) were more sporadically detected (see Table S6 in the supplemental material). Sporadically detected genes may be more sensitive indicators, whereas following up with qPCR to extract quantitative information from more commonly detected clinically relevant ARGs could be useful. Targeting key genetic elements, particularly those involved in ARG capture and dissemination, could potentially provide a more comprehensive indicator of anthropogenic ARG inputs and their potential to spread, as has been proposed in the case of class 1 integrons (42).

In the future, more comprehensive metrics derived from metagenomic data may provide a richer assessment of the true “risk” posed by a given sample in terms of the potential to spread resistance of human consequence (43). To this end, Oh et al. (44) define “relative resistome risk” as a ranking system of samples based on content of contigs containing markers corresponding to ARGs, mobile genetic elements, and pathogens (45). In the present study, it was possible to identify numerous scaffolds bearing both ARGs and mobile genetic elements, remarkably almost exclusively in conditions amended with raw manure. This finding corroborates reports by others that amending soil with manure increases the mobility of plasmids (8), taking this a step further to demonstrate the pattern on vegetables. The fact that many genes that would be expected to be colocalized were networked together provided a line of evidence of the validity of the assembly. For example, several ARGs associated with multidrug resistance, coregulatory genes, and core E. coli genes (*emrR*/*emrAB*, *baeSR*/*mdtABC*, *acrAB*) were predicted to cooccur (46). Overall, the results suggest that there is a strong potential for ARG mobility in soils receiving biological amendment and that composting could help reduce this potential.

## MATERIALS AND METHODS

### Antibiotic administration and manure collection.

Administration of antibiotics to dairy cattle was described by Ray et al. (47). Briefly, healthy lactating cows were administered standard therapeutic doses of pirlimycin (two doses of 50 mg each, 24 h apart; *n* = 3) and end-of-lactation cows received preventative doses of cephapirin (single dose of 300 mg into each of four quarters; *n* = 3). Control cows did not receive antibiotics (*n* = 3). Neither DA nor DC cows received antibiotics during the previous 12-month lactation cycle. Manure, consisting of feces and urine, was collected within the pen on days 1 to 3 from the nine cows after the start of antibiotic administration, as described by Ray et al. (47), to capture the peak antibiotic excretion period. Manure samples from the six pirlimycin- or cephapirin-treated cows were mixed to represent the DA condition, with manure from the three untreated cows collected and mixed on the same days to represent the DC condition. Manure was stored at 4°C prior to composting.

### Composting.

Manure samples were mixed with grass hay and mulch to achieve target C/N ratios and subjected to static composting (47). To accommodate multiple conditions, composting was conducted on a small scale (71-cm-length by 64-cm-diameter tumblers; *n* = 3). The target temperature of 55°C for 3 days was achieved as recommended by the Food Safety Modernization Act (48). All four compost treatments were performed in triplicate and cured for 42 days prior to storage at 4°C before applying to soils. Pirlimycin and cephapirin were detected in the DA manure used in this study, and although they were not detected after composting, metabolites were likely still present (47).

### Vegetable preparation and harvest.

Soils representing two textures were collected in Virginia (for SCL, 37° 12′ 54.5′′ N 80° 26′ 32.4′′ W; for LS, 36° 41′ 2.749′′ N 76° 46′ 5.023′′ W) (see Table S7 in the supplemental material). In each round pot (6-in. diameter, 5-in. height; Poppelmann GmgH & Co., Lohne, Germany), 0.9 kg (dry weight) of soil was mixed by hand with compost or manure to replicate the 2015 Virginia Commercial Vegetable Recommendations standard of 3 tons/acre (dry weight) (49) and then sowed with radish seeds (Raphanus sativus, Crunchy Royal; Johnny’s Seeds, Inc., Albion, ME). Lettuce seeds (Lactuca sativa, Nancy organic; Johnny’s Seeds, Inc., Albion, ME) were first sown in horticulture vermiculite (ASB Greenworld, Inc., Mattaponi, VA) to develop five or six true leaves prior to transfer to the amended soils as described above. Triplicate pots of each condition were mixed and cultivated in a greenhouse at Virginia Tech (Blacksburg, VA). Municipal water was filtered using granulated activated carbon to reduce free chlorine and chloramines, and pots were watered by hand to maintain 50 to 70% field capacity moisture. Water-soluble 20-20-20 fertilizer (JR Peters Inc., Allentown, PA) was applied to all pots as necessary to maintain plant health. Hairnets were used to cover pots during growth as a barrier to cross-contamination. Lettuce leaves and radishes were harvested after 34 days and 60 days, respectively. Gloves were worn during harvest to prevent cross-contamination. Leaves and root hairs were separated from radish taproots with ethanol-rinsed scissors and then brushed with a soft paintbrush that was rinsed with 70% ethanol and air dried between use to remove loose soil. Whole lettuce plants were first separated from the bulk soil by hand and shaking. Leaves were collected by hand for microbial DNA analysis.

10.1128/mSphere.00239-19.9 TABLE S7 Soil properties of loamy sand and silty clay loam collected in Virginia prior to seeding and mixing with any soil amendments. Analyses were performed by Waypoint Analytical Virginia, Inc. (Richmond, VA). Download Table S7, DOCX file, 0.01 MB. Copyright © 2019 Guron et al. 2019 Guron et al. This content is distributed under the terms of the Creative Commons Attribution 4.0 International license.

### DNA extraction.

Taproots or leaves were weighed in 710-ml sterile filter bags with adequate peptone buffer (Becton, Dickinson and Company, Franklin Lakes, NJ) containing Tween 80 (Fisher Scientific, Waltham, MA) (0.1% each) to create a 1/10 (wt/wt) dilution. Bacteria were removed from the vegetable surfaces by shaking at 220 rpm for 5 min using a benchtop rotator, alternating with hand-massaging for 2 min and shaking again for an additional 5 min. The suspensions were filtered through a 0.22-μm 47-mm mixed cellulose ester membrane (EMD Millipore, Burlington, MA) to collect microbial cells dissociated from the vegetable surfaces. The filters were folded, torn, transferred to Lysing Matrix E tubes (FastDNA Spin kit for Soil, MP Biomedicals, Solon, OH) and stored at −80°C until extraction. Extraction followed the manufacturer’s instructions, with the exception of an additional bead beating step and 2-h incubation (chemical lysis). DNA was eluted in 100 μl water and subjected to OneStep PCR Inhibitor Removal (Zymo Research Corporation, Irvine, CA) before storing at −80°C.

### Shotgun metagenomic sequencing.

DNA extracts from each of three independent biological replicates of 19 vegetable/soil/amendment type combinations were pooled for sequencing (134 ng each measured by NanoDrop ND-1000 spectrophotometer [Thermo Fisher Scientific, Waltham, MA]), with an additional 31 single replicates directly sequenced. This enabled assessment of whether pooling DNA, which economically generates collective DNA sequence data across multiple replicates, significantly affected the resulting sequence libraries. DNA was prepped using Accel-NGS 2S Plus DNA Library kits (Swift Biosciences, Ann Arbor, MI) and sequenced by the Biocomplexity Institute of Virginia Tech (Table S1). After adapter sequence attachment, 14 PCR cycles were performed and 1 ng of DNA from each sample was sequenced. Illumina HiSeq 2500 sequencing generated 100-bp paired-end reads, which were deposited into the Sequence Read Archive (SRP151152). Blank extracts yielded insufficient DNA for metagenomic sequencing.

### Sequencing data filtering and rarefication.

Profiling surficial vegetable microbial metagenomes is challenged by contamination with chloroplast DNA, which contains 16S rRNA genes that can be confused with bacteria. Plant-derived chloroplast sequences were downloaded from Greengenes (May 2013 release) (50) and removed using Bowtie2 (51). To account for variability in sequencing depth among the samples, the number of sequences obtained from each sample following chloroplast filtering was rarefied to 12,700,000 reads using Vsearch (52). Scripts can be found https://github.com/gaarangoa/genomic-scripts.

For the purpose of this study, ARGs were defined as gene products that are directly involved in the efflux of antibiotics, modification or inactivation of an antibiotic, or modification of the antibiotic target. Additionally, genes related to the regulation of ARGs, such as activator and sensor kinase genes, were included. Other genes in the databases, particularly those involved in “housekeeping” functions or where the wild-type genes do not confer resistance in any host, were removed from analysis. For example, genes involved in DNA replication, RNA production, protein production, or other biosynthesis processes, including *EF-Tu*, *rpoB*, and porin genes were excluded (Table S8). Additionally, *murA*, *mfd*, and *ileS* were excluded from the ARG-miner data set.

10.1128/mSphere.00239-19.10 TABLE S8 Genes from CARD and ACLAME included in this study. List of entries from CARD v1.2.1 and ACLAME v0.4 included for analysis. Download Table S8, TXT file, 0.2 MB. Copyright © 2019 Guron et al. 2019 Guron et al. This content is distributed under the terms of the Creative Commons Attribution 4.0 International license.

### Gene and taxonomy prediction.

The resulting sequence files were uploaded to MetaStorm (http://bench.cs.vt.edu/MetaStorm/) (53) and aligned to the Comprehensive Antibiotic Resistance Database (CARD v1.2.1) (14) (E value < 1e−10, identity > 80%, and ≥25 amino acids). DNA sequence reads were also compared to ARG-miner v1.1.6 (17), which includes ARGs from CARD (16), Antibiotic Resistance Genes Database (54), and UniProt, along with ARGs identified using a deep learning (deepARG) (18) algorithm and crowd-sourced annotation to keep it up to date. The relative abundance of ARGs relative to 16S rRNA gene copies, aligned to Greengenes (May 2013 release) (50) with 90% identity cutoff, was calculated as described by Li et al. (19). Taxonomic assignments for other analyses were performed using MetaPhlAn2, which takes into consideration multiple genes and not just 16S rRNA genes, and were measured in reads per kilobase per million mapped reads (55).

*De novo* assembly was performed via MetaStorm (53), with an average of 24.6% of reads successfully assembled among samples. Assembled sequences were annotated using parameters of >60% identity and >25 amino acids. Putative genes encoding functions associated with recombinase, transposase, or integrase activity were identified using ACLAME (56) (Table S8). Cooccurring genes (i.e., ≤5,000 bp apart [57]) on scaffolds were subjected to network analysis and visualized using the R packages tidygraphs and ggraph. Additional sequence visualization was performed using CLC Genomics Workbench 9.5.3, and NCBI BLASTn was used for specific alignments.

### Statistical analysis.

Analysis of similarities (ANOSIM) and nonmetric multidimensional scaling (NMDS) plots were performed using PRIMER-E (version 6.1.13) to compare resemblance data of ARG classes (Bray-Curtis). R-value cutoffs were applied as defined by Clarke and Warwick (58) (*R* > 0.75, well separated; *R* > 0.5, separated but overlapping; *R* < 0.25, barely separated). Chi-square analysis was performed with R (3.4.3) function chisq.test with Yates’ continuity correction to determine whether ARG mechanism detection depended on vegetable and soil types. Due to the lack of normal distribution, one-way comparisons of the relative abundances for each ARG class were determined using R functions for Wilcoxon test (between two levels of soil textures) or Kruskal-Wallis test (among three levels of amendment types) with pairwise comparisons using Wilcoxon and Bonferroni method for *P* value adjustment, respectively. Fold differences in relative abundances were determined using geometric means of paired treatments that were significantly different. JMP Pro 13 was used for Spearman’s correlation and for assembling stacked bar graphs. Significance was defined at *P* < 0.05.

### Ethics approval and consent to participate.

All animal studies were approved by IACUC protocol DASC 13-145 (PI, K. Knowlton).

### Availability of data and materials.

Raw reads generated and analyzed during the current study are available in the NCBI Sequence Read Archive repository (accession no. SRP151152). All fastq files were uploaded to MetaStorm (https://bench.cs.vt.edu/MetaStorm/) under projects “Greenhouse Vegetable Surfaces” (which includes all chloroplast DNA sequences) and “Greenhouse Vegetable Surfaces – corrected” (chloroplast 16S rRNA gene sequences were removed and reads were rarefied to 12,700,000). All other data generated and analyzed for this study are included as supplemental material files.
