# Supplementary material for: Effects of Dairy Manure-Based Amendments and Soil Texture on Lettuce- and Radish-Associated Microbiota and Resistomes
Source: mSphere. 2019 May 8;4(3):e00239-19. doi: 10.1128/mSphere.00239-19 (PMC6506619; doi:10.1128/mSphere.00239-19)
Supplement: TEXT S1 [file mSphere.00239-19-s0001.docx]

Supplemental Text

Title: Effect of Dairy Manure-Based Amendments and Soil Texture on Lettuce and Radish Associated Microbiota and Resistomes

Authors: Giselle K. P. Guron^a, b^, Gustavo Arango-Argoty^c^, Liqing Zhang^c^, Amy Pruden^a^, Monica A. Ponder^b^

^a^Via Department of Civil and Environmental Engineering, Virginia Tech, Blacksburg, VA, USA

^b^Department of Food Science and Technology, Virginia Tech, Blacksburg, VA, USA

^c^Department of Computer Science, Virginia Tech, Blacksburg, VA, USA

**Pooled versus individual replicate DNA sequencing comparison**

After rarefaction, there were no significant differences in total ARGs identified through comparison to CARD between the pooled and individual lettuce samples, except tetracenomycin ARGs, which were 2.8-fold greater in individual than in pooled lettuce samples grown in LS (p<0.033). In contrast, pooled radish samples contained 1.5-fold greater total ARGs on average relative to individual samples (p<0.008).

**Comparison of deep-learning versus similarity cut-off–based ARG identification.**

When comparing lettuce conditions, ARG-miner detected differences not noted when the data were compared against CARD. For example, kasugamycin ARGs are not included in CARD, while ARG-miner detected 6-fold greater abundance of this class of ARGs on lettuce grown in manure-amended LS compared to the same amendment added to silty clay loam (SCL) (p<0.032) (Fig. 1b). According to ARG-miner, lettuce grown in compost-amended LS carried greater fosfomycin (4.1-fold), sulfonamide (2.4-fold), and tetracenomycin (3.1-fold) ARG classes than lettuce grown in compost-amended SCL (p<0.032), differences which were not reflected by comparison to the CARD database. Still, comparison to CARD did indicate some differences not detected by ARG-miner. For example, comparison to CARD indicated that aminoglycoside ARGs were greater on lettuce grown in manure-amended than in compost-amended SCL ARG-miner. However, sulfonamide ARGs from manure-amended lettuce were 4.0-fold greater than that of the compost-amended lettuce in SCL (p<0.013) when analyzed using ARG-miner only.

For radishes, the ARG-miner pipeline indicated that manure-amendment resulted in 2.1-fold greater phenicol ARGs than compost-amendment when grown in SCL (p<0.013), whereas CARD did not indicate any effects of amendment type on individual ARG classes carried. In terms of effects of soil texture, analysis using the ARG-miner pipeline indicated that radishes grown in manure-amended SCL had greater fosmidomycin (3.9-fold, p<0.0381), kasugamycin (3.9-fold, p<0.038), phenicol (2.0-fold, p<0.019), and quinoline (2.0-fold, p<0.038) ARGs than when grown in manure-amended LS, but the greater peptide ARGs indicated above by comparison to CARD was not found. Similarly, radishes grown in compost-amended SCL carried 1.4-fold greater tetracycline ARGs (p<0.038) than when grown in compost-amended LS, which was not noted using CARD. On the other hand, ARG-miner indicated that bacitracin ARGs were 1.6-fold greater on radishes from manure-amended LS than manure-amended SCL (p<0.010). Amendment of compost to LS also resulted in greater pleuromutilin ARGs (1.5-fold) than when amended to SCL (p<0.010), which was consistent with CARD results, but the greater sulfonamide and tetracenomycin ARGs noted by comparison to CARD where not found when analyzed using ARG-miner.
