# Supplementary material for: Effects of Dairy Manure-Based Amendments and Soil Texture on Lettuce- and Radish-Associated Microbiota and Resistomes
Source: mSphere. 2019 May 8;4(3):e00239-19. doi: 10.1128/mSphere.00239-19 (PMC6506619; doi:10.1128/mSphere.00239-19)
Supplement: TABLE S4 [file mSphere.00239-19-st004.docx]

|  |  |  |  |  |  | ARGs |  |  |  | Taxonomy | |  |  |
| --- | --- | --- | --- | --- | --- | --- | --- | --- | --- | --- | --- | --- | --- |
| Sample | Vegetable | Soil Texture | Amendment Type | Cattle Type | Reps | Richness | *H_max_ | **H’ | ***E | Richness | *H_max_ | **H’ | ***E |
| L10DM-L11DM-L12DM | Lettuce | Silty Clay Loam | Manure | DC | 3 | 196 | 5.28 | 4.21 | 0.8 | 57 | 4.04 | 1.78 | 0.44 |
| L11DM | Lettuce | Silty Clay Loam | Manure | DC | 1 | 144 | 4.97 | 4.42 | 0.89 | 22 | 3.09 | 2.26 | 0.73 |
| L12DM | Lettuce | Silty Clay Loam | Manure | DC | 1 | 198 | 5.29 | 4.13 | 0.78 | 48 | 3.87 | 1.75 | 0.45 |
| L14DM | Lettuce | Silty Clay Loam | Manure | DA | 1 | 144 | 4.97 | 4.3 | 0.87 | 33 | 3.5 | 2.35 | 0.67 |
| L14DM-L15DM-L16DM | Lettuce | Silty Clay Loam | Manure | DA | 3 | 223 | 5.41 | 4.24 | 0.78 | 49 | 3.89 | 3.25 | 0.83 |
| L15DM | Lettuce | Silty Clay Loam | Manure | DA | 1 | 229 | 5.43 | 4.22 | 0.78 | 44 | 3.78 | 3.19 | 0.84 |
| L17D-L18D-L19D | Lettuce | Silty Clay Loam | Compost | DC | 3 | 138 | 4.93 | 4.09 | 0.83 | 30 | 3.4 | 2.13 | 0.63 |
| L18D | Lettuce | Silty Clay Loam | Compost | DC | 1 | 115 | 4.74 | 3.63 | 0.77 | 25 | 3.22 | 2.45 | 0.76 |
| L19D | Lettuce | Silty Clay Loam | Compost | DC | 1 | 89 | 4.49 | 3.87 | 0.86 | 12 | 2.48 | 1.47 | 0.59 |
| L1D-L2D-L3D | Lettuce | Loamy Sand | Compost | DC | 3 | 295 | 5.69 | 4.61 | 0.81 | 83 | 4.42 | 2.3 | 0.52 |
| L1DM-L2DM-L3DM | Lettuce | Loamy Sand | Manure | DC | 3 | 275 | 5.62 | 4.66 | 0.83 | 77 | 4.34 | 2.66 | 0.61 |
| L21D | Lettuce | Silty Clay Loam | Compost | DA | 1 | 148 | 5 | 3.85 | 0.77 | 52 | 3.95 | 2.68 | 0.68 |
| L21D-L22D-L23D | Lettuce | Silty Clay Loam | Compost | DA | 3 | 153 | 5.03 | 3.95 | 0.79 | 54 | 3.99 | 3.11 | 0.78 |
| L23D | Lettuce | Silty Clay Loam | Compost | DA | 1 | 146 | 4.98 | 3.7 | 0.74 | 45 | 3.81 | 2.69 | 0.71 |
| L2D | Lettuce | Loamy Sand | Compost | DC | 1 | 302 | 5.71 | 4.56 | 0.8 | 86 | 4.45 | 2.26 | 0.51 |
| L2F | Lettuce | Loamy Sand | Fertilizer Control | Fertilizer Control | 1 | 215 | 5.37 | 4.28 | 0.8 | 66 | 4.19 | 2.96 | 0.71 |
| L2F-L3F-L4F | Lettuce | Loamy Sand | Fertilizer Control | Fertilizer Control | 3 | 183 | 5.21 | 3.97 | 0.76 | 45 | 3.81 | 2.84 | 0.75 |
| L3DM | Lettuce | Loamy Sand | Manure | DC | 1 | 252 | 5.53 | 4.58 | 0.83 | 67 | 4.2 | 2.93 | 0.7 |
| L5D | Lettuce | Loamy Sand | Compost | DA | 1 | 288 | 5.66 | 4.61 | 0.81 | 77 | 4.34 | 2.76 | 0.64 |
| L5D-L7D-L8D | Lettuce | Loamy Sand | Compost | DA | 3 | 280 | 5.63 | 4.54 | 0.81 | 77 | 4.34 | 2.78 | 0.64 |
| L5F | Lettuce | Silty Clay Loam | Fertilizer Control | Fertilizer Control | 1 | 151 | 5.02 | 3.85 | 0.77 | 41 | 3.71 | 3.02 | 0.81 |
| L6DM-L7DM-L8DM | Lettuce | Loamy Sand | Manure | DA | 3 | 264 | 5.58 | 4.67 | 0.84 | 67 | 4.2 | 3.05 | 0.72 |
| L6F | Lettuce | Silty Clay Loam | Fertilizer Control | Fertilizer Control | 1 | 173 | 5.15 | 3.85 | 0.75 | 58 | 4.06 | 2.67 | 0.66 |
| L7F | Lettuce | Silty Clay Loam | Fertilizer Control | Fertilizer Control | 1 | 177 | 5.18 | 3.83 | 0.74 | 56 | 4.03 | 3.22 | 0.8 |
| L8DM | Lettuce | Loamy Sand | Manure | DA | 1 | 174 | 5.16 | 4.52 | 0.88 | 42 | 3.74 | 2.21 | 0.59 |
| R11DM | Radish | Silty Clay Loam | Manure | DC | 3 | 303 | 5.71 | 4.55 | 0.8 | 71 | 4.26 | 2.67 | 0.63 |
| R13DM | Radish | Silty Clay Loam | Manure | DA | 1 | 280 | 5.63 | 4.6 | 0.82 | 77 | 4.34 | 2.5 | 0.58 |
| R13DM-R14DM-R15DM | Radish | Silty Clay Loam | Manure | DA | 3 | 300 | 5.7 | 4.63 | 0.81 | 84 | 4.43 | 2.63 | 0.59 |
| R14DM | Radish | Silty Clay Loam | Manure | DA | 1 | 275 | 5.62 | 4.56 | 0.81 | 69 | 4.23 | 2.42 | 0.57 |
| R17D | Radish | Silty Clay Loam | Compost | DC | 3 | 309 | 5.73 | 4.62 | 0.81 | 76 | 4.33 | 2.9 | 0.67 |
| R17D-R18D-R19D | Radish | Silty Clay Loam | Compost | DC | 1 | 328 | 5.79 | 4.55 | 0.79 | 85 | 4.44 | 3.44 | 0.77 |
| R19D | Radish | Silty Clay Loam | Compost | DC | 3 | 305 | 5.72 | 4.38 | 0.77 | 84 | 4.43 | 3.35 | 0.76 |
| R1D | Radish | Loamy Sand | Compost | DC | 1 | 318 | 5.76 | 4.66 | 0.81 | 98 | 4.58 | 2.76 | 0.6 |
| R1D-R2D-R3D | Radish | Loamy Sand | Compost | DC | 3 | 341 | 5.83 | 4.66 | 0.8 | 100 | 4.61 | 3.02 | 0.66 |
| R21D-R22D-R23D | Radish | Silty Clay Loam | Compost | DA | 3 | 309 | 5.73 | 4.38 | 0.76 | 92 | 4.52 | 3.04 | 0.67 |
| R22D | Radish | Silty Clay Loam | Compost | DA | 1 | 266 | 5.58 | 4.03 | 0.72 | 67 | 4.2 | 2.78 | 0.66 |
| R23D | Radish | Silty Clay Loam | Compost | DA | 1 | 275 | 5.62 | 4.24 | 0.76 | 79 | 4.37 | 3.03 | 0.69 |
| R2DM | Radish | Loamy Sand | Manure | DC | 1 | 256 | 5.55 | 4.38 | 0.79 | 58 | 4.06 | 2.6 | 0.64 |
| R2DM-R3DM-R4DM | Radish | Loamy Sand | Manure | DC | 3 | 240 | 5.48 | 4.32 | 0.79 | 70 | 4.25 | 2.33 | 0.55 |
| R2F-R3F-R4F | Radish | Loamy Sand | Fertilizer Control | Fertilizer Control | 3 | 260 | 5.56 | 4.26 | 0.77 | 67 | 4.2 | 3.13 | 0.74 |
| R3F | Radish | Loamy Sand | Fertilizer Control | Fertilizer Control | 1 | 266 | 5.58 | 4.21 | 0.75 | 66 | 4.19 | 2.92 | 0.7 |
| R5D-R6D-R7D | Radish | Loamy Sand | Compost | DA | 3 | 298 | 5.7 | 4.56 | 0.8 | 93 | 4.53 | 3.15 | 0.7 |
| R6D | Radish | Loamy Sand | Compost | DA | 1 | 310 | 5.74 | 4.57 | 0.8 | 90 | 4.5 | 3.25 | 0.72 |
| R6DM-R7DM-R8DM | Radish | Loamy Sand | Manure | DA | 3 | 295 | 5.69 | 4.43 | 0.78 | 77 | 4.34 | 2.9 | 0.67 |
| R6F | Radish | Silty Clay Loam | Fertilizer Control | Fertilizer Control | 1 | 234 | 5.46 | 4.07 | 0.75 | 72 | 4.28 | 3 | 0.7 |
| R6F-R7F-R8F | Radish | Silty Clay Loam | Fertilizer Control | Fertilizer Control | 3 | 262 | 5.57 | 4.09 | 0.74 | 74 | 4.3 | 3.26 | 0.76 |
| R7F | Radish | Silty Clay Loam | Fertilizer Control | Fertilizer Control | 1 | 249 | 5.52 | 4.12 | 0.75 | 66 | 4.19 | 3.01 | 0.72 |
| R8DM | Radish | Loamy Sand | Manure | DA | 1 | 275 | 5.62 | 4.44 | 0.79 | 71 | 4.26 | 2.64 | 0.62 |
| R9DM | Radish | Silty Clay Loam | Manure | DC | 1 | 300 | 5.7 | 4.35 | 0.76 | 73 | 4.29 | 2.94 | 0.68 |
| R9DM-R10DM-R11DM | Radish | Silty Clay Loam | Manure | DC | 3 | 312 | 5.74 | 4.61 | 0.8 | 83 | 4.42 | 3.04 | 0.69 |

*H_max_ = ln (#ARGs or species)

**Shannon: H'=-∑[(n1/N)ln(n1/N)]

***Evenness: E=H'/H_max_
