# Supplementary material for: Effects of Dairy Manure-Based Amendments and Soil Texture on Lettuce- and Radish-Associated Microbiota and Resistomes
Source: mSphere. 2019 May 8;4(3):e00239-19. doi: 10.1128/mSphere.00239-19 (PMC6506619; doi:10.1128/mSphere.00239-19)
Supplement: FIG S1 [file mSphere.00239-19-sf001.pdf]

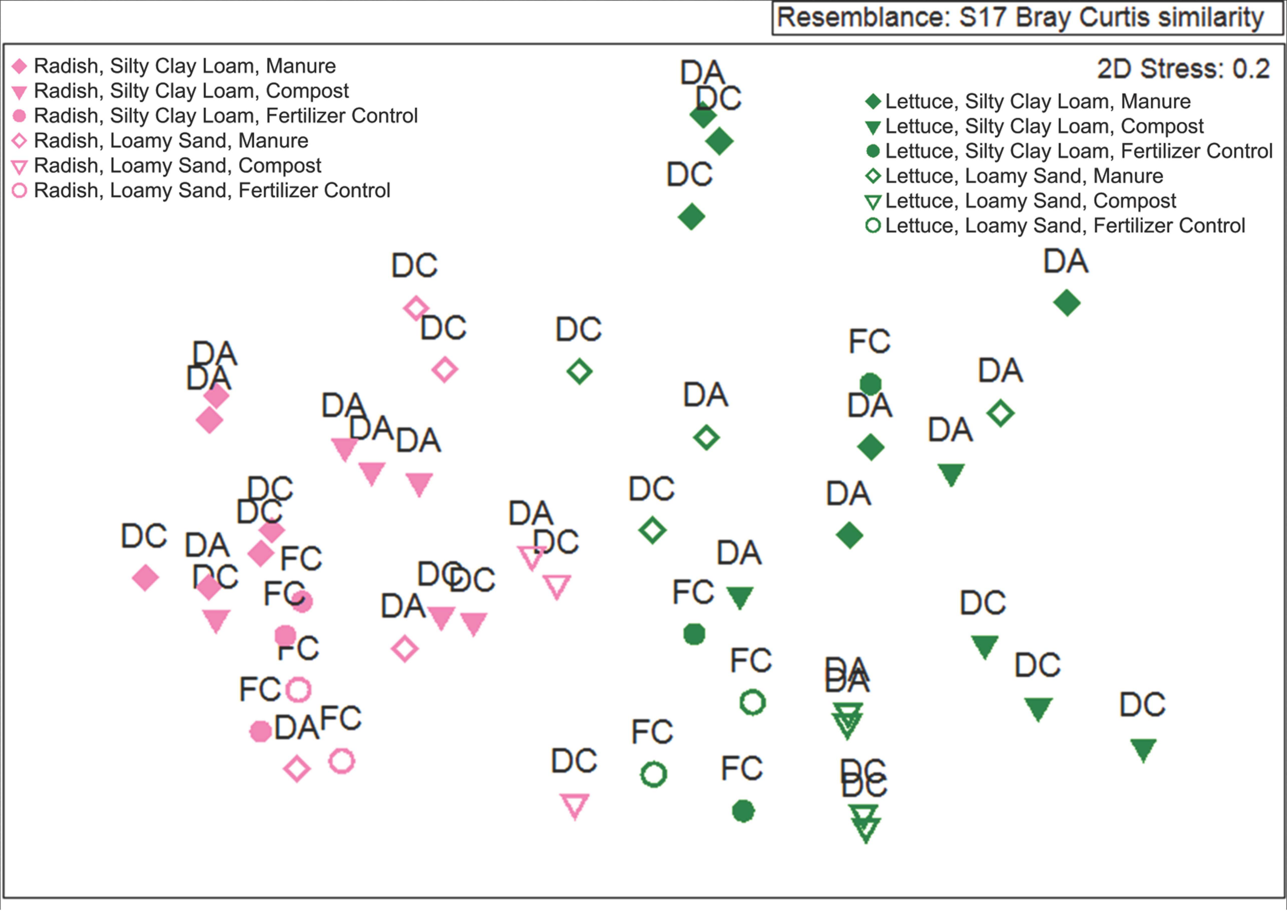
Resemblance: S17 Bray Curtis similarity
2D Stress: 0.2
Radish, Silty Clay Loam, Manure
Radish, Silty Clay Loam, Compost
Radish, Silty Clay Loam, Fertilizer Control
Radish, Loamy Sand, Manure
Radish, Loamy Sand, Compost
Radish, Loamy Sand, Fertilizer Control
Lettuce, Silty Clay Loam, Manure
Lettuce, Silty Clay Loam, Compost
Lettuce, Silty Clay Loam, Fertilizer Control
Lettuce, Loamy Sand, Manure
Lettuce, Loamy Sand, Compost
Lettuce, Loamy Sand, Fertilizer Control
DA
DC
FC
