# Supplementary material for: Effects of Dairy Manure-Based Amendments and Soil Texture on Lettuce- and Radish-Associated Microbiota and Resistomes
Source: mSphere. 2019 May 8;4(3):e00239-19. doi: 10.1128/mSphere.00239-19 (PMC6506619; doi:10.1128/mSphere.00239-19)
Supplement: TABLE S5 [file mSphere.00239-19-st005.docx]

|  | Lettuce |  |  |  |  |  |  |  |  |  |
| --- | --- | --- | --- | --- | --- | --- | --- | --- | --- | --- |
|  | DA Manure | | DA Compost | | DC Manure | | DC Compost | | Fertilizer Control | |
|  | LS (n=2) | SCL (n=3) | LS (n=2) | SCL (n=3) | LS (n=2) | SCL (n=3) | LS (n=2) | SCL (n=3) | LS (n=2) | SCL (n=3) |
| *Proteobacteria* | 42.77 | 41.6 | 22.73 | 42.97 | 66.41 | 49.11 | 23.8 | 5.77 | 37.23 | 23.27 |
| *Pseudomonadaceae* | 4.62 | 16.03 | 11.43 | 1.7 | 42.1 | 38.38 | 18.68 | 1.83 | 2.91 | 3.24 |
| *Sphingomonadaceae* | 2.33 | 3.56 | 2.13 | 5.68 | 2.28 | 0.46 | 1.79 | 0.73 | 4.29 | 3.74 |
| *Comamonadaceae* | nd | 1.4 | 0.09 | 1.25 | 1.08 | 2.38 | 0.07 | 0.6 | 1.55 | 1.42 |
| *Caulobacteraceae* | 2.18 | 1.61 | 1.25 | 32.1 | 4.73 | 0.22 | 1.01 | 0.84 | 13.13 | 2.95 |
| *Rhizobiaceae* | 0.96 | nd | 0.31 | 2.46 | 0.51 | nd | 0.17 | nd | 0.07 | 1.14 |
| *Bradyrhizobiaceae* | 1.36 | 4.37 | 1.59 | 2.14 | 2.83 | 0.16 | 0.45 | 2.5 | 3.38 | 1.76 |
| *Methylophilaceae* | nd | nd | 0.6 | 1.87 | 1.44 | nd | 0.09 | nd | 2.07 | 1 |
| *Enterobacteriaceae* | 33.12 | nd | nd | nd | 2.25 | 0.51 | 0.08 | nd | nd | nd |
| *Actinobacteria* | 41.83 | 35.63 | 49.26 | 37.55 | 16.62 | 24.29 | 62.21 | 58.69 | 44.95 | 50.69 |
| *Propionibacteriaceae* | 26.37 | 14.59 | 1.51 | 15.56 | 1.31 | 19.78 | 0.68 | 3.26 | 6.05 | 7.71 |
| *Pseudonocardiaceae* | 4.5 | 1.28 | 15.75 | 4.55 | 0.8 | 0.2 | 18.2 | 11.79 | 1.12 | 4.9 |
| Unclassified *Solirubrobacterales* | 0.46 | 8.18 | 0.73 | 2.78 | 2.49 | 0.43 | 0.21 | 2.05 | 3.39 | 6.4 |
| *Micrococcaceae* | 2.05 | 1.42 | 0.25 | 1.44 | 0.36 | 3.15 | 0.04 | 5.36 | 0.25 | 1 |
| *Microbacteriaceae* | 4.48 | 2.54 | 0.69 | 1.68 | 2.79 | 0.25 | 0.31 | 0.34 | 0.99 | 3 |
| *Nocardioidaceae* | 1.22 | 2.73 | 1.43 | 2.1 | 1.78 | 0.24 | 0.84 | 1.15 | 19.84 | 18.33 |
| *Nocardiopsaceae* | 3.04 | 1.05 | 25.53 | 6.31 | 1.36 | 0.19 | 38.39 | 34.16 | 10.49 | 3.47 |
| *Bacteroidetes* | 3.14 | nd | 0.09 | 0.61 | 1.12 | nd | 0.34 | nd | 0.16 | 0.61 |
| *Sphingobacteriaceae* | 2.78 | nd | nd | 0.61 | 0.85 | nd | 0.28 | nd | nd | 0.61 |
| *Flavobacteriaceae* | 0.36 | nd | nd | nd | 0.05 | nd | 0.04 | nd | nd | nd |
| *Firmicutes* | 7.25 | 4.6 | 26.62 | 4.46 | 11.79 | 15.22 | 13.15 | 8.03 | 10.58 | 3.75 |
| *Bacillaceae* | 5.91 | 3.68 | 15.14 | 1.43 | 11.54 | 15.15 | 5.02 | 2.6 | 8.62 | 3.22 |
| *Planococcaeae* | 0.51 | 0.07 | 9.25 | 2.37 | 0.21 | nd | 6.83 | 4.53 | 1.49 | 0.14 |
| Viruses | 4.01 | 12.08 | 0.82 | 11.35 | 1.76 | 10.5 | 0.42 | 26.52 | 4.28 | 10.75 |
| *Potyviridae* | 1.88 | 10.21 | 0.82 | 6.95 | 0.98 | 6.58 | 0.2 | 18.48 | 3.29 | 6.43 |

|  | Radish |  |  |  |  |  |  |  |  |  |
| --- | --- | --- | --- | --- | --- | --- | --- | --- | --- | --- |
|  | DA Manure | | DA Compost | | DC Manure | | DC Compost | | Fertilizer Control | |
|  | LS (n=2) | SCL (n=3) | LS (n=2) | SCL (n=3) | LS (n=2) | SCL (n=3) | LS (n=2) | SCL (n=3) | LS (n=2) | SCL (n=3) |
| *Proteobacteria* | 81 | 61.32 | 62.39 | 71.21 | 89.61 | 69.46 | 49.03 | 57.35 | 80.33 | 64.12 |
| *Pseudomonadaceae* | 9.96 | 16.97 | 13.64 | 21.88 | 36.76 | 6.29 | 12.56 | 7.6 | 1.94 | 3.88 |
| *Sphingomonadaceae* | 35.09 | 3.73 | 12.17 | 4.43 | 3.71 | 5.53 | 3.81 | 2.97 | 28.59 | 15.17 |
| *Comamonadaceae* | 3.98 | 4.62 | 9.55 | 12.55 | 2.21 | 8.14 | 18.26 | 9.22 | 8.66 | 9.83 |
| *Caulobacteraceae* | 12.32 | 2.56 | 14.67 | 7.72 | 14.15 | 2.97 | 6.54 | 6.81 | 21.69 | 9.06 |
| *Rhizobiaceae* | 1.93 | 23.98 | 2.67 | 18.68 | 2.49 | 27.87 | 0.87 | 18.2 | 6.27 | 11.9 |
| *Bradyrhizobiaceae* | 3.61 | 0.34 | 2.11 | 0.43 | 3.38 | 0.76 | 1.11 | 0.65 | 3.42 | 1.08 |
| *Methylophilaceae* | 0.95 | 0.31 | 0.4 | 0.47 | 20.79 | 0.24 | 0.18 | 0.69 | 2 | 3.22 |
| *Enterobacteriaceae* | 1.79 | 4.89 | nd | 1.35 | 0.04 | 14.88 | 0.18 | 7.96 | nd | 0.4 |
| *Actinobacteria* | 7.85 | 24.94 | 13.63 | 11.25 | 6.42 | 13.57 | 34.15 | 17.45 | 8.05 | 11.01 |
| *Propionibacteriaceae* | 0.64 | 10.7 | 0.4 | 0.49 | 0.78 | 7.15 | 0.19 | 1.17 | 1.47 | 1.59 |
| *Pseudonocardiaceae* | 0.29 | 0.63 | 3.62 | 1.36 | 0.33 | 0.19 | 8.09 | 3.57 | 1.87 | 1.2 |
| Unclassified *Solirubrobacterales* | 1.86 | 0.23 | 0.37 | 0.51 | 0.03 | 0.72 | 0.22 | 0.48 | 0.69 | 0.95 |
| *Micrococcaceae* | nd | 14.88 | nd | 2.67 | 0.05 | 0.26 | 1.73 | 0.69 | nd | 2.34 |
| *Microbacteriaceae* | 3.21 | 1.5 | 2.56 | 1.51 | 5.12 | 2.8 | 1.05 | 1.85 | 1.57 | 1.4 |
| *Nocardioidaceae* | 0.57 | 0.23 | 0.29 | 2.16 | 0.04 | 0.67 | 0.18 | 1.64 | 0.42 | 1.93 |
| *Nocardiopsaceae* | nd | nd | 4.89 | 1.51 | nd | nd | 20.62 | 6.47 | nd | nd |
| *Bacteroidetes* | 9.92 | 12.73 | 7.01 | 14.3 | 1.8 | 15.15 | 5.02 | 20.42 | 9.3 | 23.07 |
| *Sphingobacteriaceae* | 7.82 | 11.37 | 0.74 | 8.36 | 1.63 | 11.04 | 4.1 | 10.78 | 4.6 | 21 |
| *Flavobacteriaceae* | 0.02 | 0.79 | 5.97 | 4.79 | 0.02 | 3.31 | 0.64 | 8.96 | 3.39 | 1.1 |
| *Firmicutes* | 0.98 | 0.72 | 16.58 | 2.86 | 1.98 | 1.18 | 11.59 | 4.48 | 1.19 | 0.09 |
| *Bacillaceae* | 0.98 | 0.68 | 13.54 | 2.16 | 1.98 | 1.13 | 6.01 | 3.19 | 1.19 | 0.09 |
| *Planococcaeae* | nd | nd | 2.54 | 0.63 | nd | nd | 4.62 | 1.05 | nd | nd |
| Viruses | 0.19 | 0.24 | 0.31 | 0.19 | 0.18 | 0.57 | 0.03 | 0.2 | 0.49 | 0.85 |
| *Potyviridae* | 0.19 | 0.23 | 0.28 | 0.19 | 0.18 | 0.56 | 0.03 | 0.11 | 0.49 | 0.7 |

nd: not detected
