# Supplementary material for: Effects of Dairy Manure-Based Amendments and Soil Texture on Lettuce- and Radish-Associated Microbiota and Resistomes
Source: mSphere. 2019 May 8;4(3):e00239-19. doi: 10.1128/mSphere.00239-19 (PMC6506619; doi:10.1128/mSphere.00239-19)
Supplement: TABLE S6 [file mSphere.00239-19-st006.docx]

|  |  | Selected beta-lactamase genes | | | |  |  |  |  |  |  |
| --- | --- | --- | --- | --- | --- | --- | --- | --- | --- | --- | --- |
| Lettuce |  | *OXA* | *CTX-M* | *SHV* | *TEM* | *CMY* | *KPC* | *VEB* | *PER* | *GES* | *SME* |
| Loamy Sand | DA Manure (n=2) | 0.0124 | 0.0016 | nd | nd | nd | nd | nd | 0.0015 | nd | nd |
|  | DC Manure (n=2) | 0.0041 | nd | nd | nd | 0.0023 | nd | nd | 0.0014 | nd | nd |
|  | DA Compost (n=2) | 0.0066 | 0.0031 | nd | 0.005 | 0.0019 | nd | 0.0012 | nd | nd | nd |
|  | DC Compost (n=2) | 0.0043 | nd | nd | nd | 0.0008 | 0.002 | nd | nd | nd | nd |
|  | Fertilizer Control (n=2) | 0.004 | 0.0024 | 0.0012 | 0.0043 | nd | 0.0012 | nd | nd | nd | nd |
| Silty Clay Loam | DA Manure (n=2) | 0.0036 | 0.0023 | nd | nd | nd | nd | nd | nd | nd | nd |
|  | DC Manure (n=2) | 0.0071 | 0.0044 | nd | 0.0039 | nd | 0.0017 | nd | nd | nd | nd |
|  | DA Compost (n=2) | 0.0066 | nd | 0.0028 | 0.0028 | nd | nd | nd | nd | nd | nd |
|  | DC Compost (n=2) | 0.0046 | nd | nd | nd | nd | nd | nd | nd | nd | nd |
|  | Fertilizer Control (n=2) | 0.0065 | 0.0026 | 0.0026 | 0.0045 | nd | nd | nd | nd | nd | nd |
| Radish |  |  |  |  |  |  |  |  |  |  |  |
| Loamy Sand | DA Manure (n=3) | 0.0124 | 0.003 | nd | 0.0016 | nd | nd | nd | nd | 0.0015 | nd |
|  | DC Manure (n=3) | 0.0057 | 0.003 | 0.0021 | nd | 0.0008 | nd | nd | nd | nd | nd |
|  | DA Compost (n=3) | 0.005 | 0.0035 | 0.0152 | 0.0015 | 0.0049 | 0.002 | nd | nd | nd | nd |
|  | DC Compost (n=3) | 0.008 | 0.007 | nd | nd | nd | 0.001 | nd | nd | nd | nd |
|  | Fertilizer Control (n=3) | 0.0123 | 0.0033 | 0.0014 | nd | 0.0015 | 0.0013 | nd | nd | nd | nd |
| Silty Clay Loam | DA Manure (n=3) | 0.0131 | 0.0055 | 0.0012 | nd | 0.002 | 0.0032 | nd | nd | nd | nd |
|  | DC Manure (n=3) | 0.0068 | 0.003 | 0.0047 | 0.0018 | 0.0044 | 0.0013 | 0.0012 | nd | nd | 0.0016 |
|  | DA Compost (n=3) | 0.0095 | nd | 0.0028 | nd | 0.0009 | nd | nd | nd | nd | nd |
|  | DC Compost (n=3) | 0.0069 | 0.0025 | 0.0026 | 0.0012 | 0.0032 | 0.0014 | nd | nd | nd | nd |
|  | Fertilizer Control (n=3) | 0.011 | nd | 0.0041 | 0.0016 | 0.0012 | 0.0017 | 0.0015 | nd | nd | nd |

|  |  | Selected MLS ARGs | | Colistin ARGs | Quinolone ARGs | |
| --- | --- | --- | --- | --- | --- | --- |
| Lettuce |  | *erm* | *lnu* | *mcr*-like genes | *qnrB* variants | *qnrD* variants |
| Loamy Sand | DA Manure (n=2) | 0.0206 | 0.0087 | nd | 0.003 | nd |
|  | DC Manure (n=2) | 0.0133 | nd | nd | 0.0018 | nd |
|  | DA Compost (n=2) | 0.0236 | 0.0041 | 0.0007 | nd | nd |
|  | DC Compost (n=2) | 0.0238 | 0.0032 | 0.0005 | nd | nd |
|  | Fertilizer Control (n=2) | 0.0172 | 0.0144 | 0.0034 | nd | nd |
| Silty Clay Loam | DA Manure (n=2) | 0.0094 | 0.0198 | nd | nd | nd |
|  | DC Manure (n=2) | 0.0096 | 0.003 | nd | nd | nd |
|  | DA Compost (n=2) | 0.0076 | 0.005 | 0.0015 | nd | nd |
|  | DC Compost (n=2) | 0.0072 | 0.0053 | 0.0015 | nd | nd |
|  | Fertilizer Control (n=2) | 0.007 | 0.0045 | nd | nd | nd |
| Radish |  |  |  |  |  |  |
| Loamy Sand | DA Manure (n=3) | 0.0056 | 0.0017 | 0.0041 | nd | nd |
|  | DC Manure (n=3) | 0.0068 | nd | nd | nd | nd |
|  | DA Compost (n=3) | 0.0103 | nd | 0.0046 | 0.0013 | nd |
|  | DC Compost (n=3) | 0.0191 | 0.0022 | 0.0011 | nd | nd |
|  | Fertilizer Control (n=3) | 0.0089 | nd | 0.0007 | nd | nd |
| Silty Clay Loam | DA Manure (n=3) | 0.0195 | 0.0011 | 0.0012 | 0.0051 | 0.0014 |
|  | DC Manure (n=3) | 0.0102 | nd | 0.0012 | 0.0741 | 0.0048 |
|  | DA Compost (n=3) | 0.0099 | 0.0014 | 0.0013 | 0.0015 | 0.0033 |
|  | DC Compost (n=3) | 0.0082 | 0.0015 | 0.0009 | 0.0382 | 0.0028 |
|  | Fertilizer Control (n=3) | 0.0082 | nd | 0.0013 | 0.0035 | 0.0018 |

|  |  | *vanHAX*-like gene cluster | | | *vanC(XY)T* gene cluster | | |
| --- | --- | --- | --- | --- | --- | --- | --- |
| Lettuce |  | Dehydrogenase gene | D-Ala-D-Lac ligase gene | D,D-dipeptidase gene | D-Ala-D-Ser ligase gene | D,D-dipeptidase gene | Serine racemase gene |
| Loamy Sand | DA Manure (n=2) | 0.0061 | 0.0088 | 0.0103 | nd | nd | nd |
|  | DC Manure (n=2) | 0.0046 | 0.0109 | 0.0019 | 0.0011 | nd | 0.0011 |
|  | DA Compost (n=2) | 0.0094 | 0.0185 | 0.0221 | 0.0041 | nd | nd |
|  | DC Compost (n=2) | 0.0244 | 0.0441 | 0.0552 | 0.0056 | 0.003 | nd |
|  | Fertilizer Control (n=2) | 0.0114 | 0.0139 | 0.0177 | 0.0017 | nd | nd |
| Silty Clay Loam | DA Manure (n=2) | 0.0034 | 0.0263 | 0.0125 | 0.0019 | nd | nd |
|  | DC Manure (n=2) | nd | 0.0041 | 0.0044 | 0.0042 | nd | nd |
|  | DA Compost (n=2) | 0.0025 | 0.0074 | 0.0086 | 0.0046 | nd | nd |
|  | DC Compost (n=2) | 0.0075 | 0.0104 | 0.0053 | 0.0022 | nd | nd |
|  | Fertilizer Control (n=2) | 0.0089 | 0.0246 | 0.0098 | nd | nd | nd |
| Radish |  |  |  |  |  |  |  |
| Loamy Sand | DA Manure (n=3) | 0.004 | 0.0103 | 0.0152 | nd | nd | nd |
|  | DC Manure (n=3) | 0.0013 | 0.0032 | 0.0016 | nd | nd | nd |
|  | DA Compost (n=3) | 0.0095 | 0.0197 | 0.0152 | 0.0017 | nd | nd |
|  | DC Compost (n=3) | 0.0199 | 0.0371 | 0.0288 | 0.0033 | 0.0041 | nd |
|  | Fertilizer Control (n=3) | 0.0081 | 0.0186 | 0.0096 | nd | nd | nd |
| Silty Clay Loam | DA Manure (n=3) | 0.0045 | 0.016 | 0.0169 | 0.0021 | nd | nd |
|  | DC Manure (n=3) | 0.0065 | 0.0276 | 0.0259 | 0.002 | nd | nd |
|  | DA Compost (n=3) | 0.0145 | 0.0225 | 0.0279 | 0.0035 | 0.0017 | nd |
|  | DC Compost (n=3) | 0.0092 | 0.0285 | 0.0221 | 0.0039 | nd | nd |
|  | Fertilizer Control (n=3) | 0.0068 | 0.0201 | 0.0144 | 0.0028 | nd | nd |

nd: not detected
