# Supplementary material for: Effects of Dairy Manure-Based Amendments and Soil Texture on Lettuce- and Radish-Associated Microbiota and Resistomes
Source: mSphere. 2019 May 8;4(3):e00239-19. doi: 10.1128/mSphere.00239-19 (PMC6506619; doi:10.1128/mSphere.00239-19)
Supplement: TABLE S7 [file mSphere.00239-19-st007.docx]

|  |  |  |  |  |  |  |  |  |  | Percent Base Saturation | | | | |
| --- | --- | --- | --- | --- | --- | --- | --- | --- | --- | --- | --- | --- | --- | --- |
| Soil Texture | Organic Matter (% Rate) | Estimated Nitrogen Release | P (ppm, Mehlich 3) | K (ppm) | Mg (ppm) | Ca (ppm) | pH | Acidity (mEq/100 g) | Cation Exchange Capacity (mEq/100 g) | K (%) | Mg (%) | Ca (%) | H (%) | NO3N (ppm) |
| Silty Clay Loam | 8.6 | 150 | 75 | 340 | 522 | 2074 | 6.8 | 0.5 | 16.1 | 5.4 | 27 | 64.4 | 2.9 | 37 |
| Loamy Sand | 1.5 | 75 | 198 | 165 | 47 | 305 | 5.3 (buffer index 6.82) | 1.1 | 3.4 | 12.4 | 11.5 | 44.9 | 31 | 8 |
